# Amyloid Precursor Protein and Alzheimer’s Disease

**DOI:** 10.3390/ijms241914794

**Published:** 2023-09-30

**Authors:** Kseniia S. Orobets, Andrey L. Karamyshev

**Affiliations:** Department of Cell Biology and Biochemistry, Texas Tech University Health Sciences Center, Lubbock, TX 79430, USA; korobets@ttuhsc.edu

**Keywords:** neurodegenerative disease, Alzheimer’s disease, amyloid precursor protein (APP), amyloid beta, protein biogenesis, protein transport, membrane proteins, SRP-dependent targeting

## Abstract

Alzheimer’s disease (AD) is one of the most common neurodegenerative disorders associated with age or inherited mutations. It is characterized by severe dementia in the late stages that affect memory, cognitive functions, and daily life overall. AD progression is linked to the accumulation of cytotoxic amyloid beta (Aβ) and hyperphosphorylated tau protein combined with other pathological features such as synaptic loss, defective energy metabolism, imbalances in protein, and metal homeostasis. Several treatment options for AD are under investigation, including antibody-based therapy and stem cell transplantation. Amyloid precursor protein (APP) is a membrane protein considered to play a main role in AD pathology. It is known that APP in physiological conditions follows a non-amyloidogenic pathway; however, it can proceed to an amyloidogenic scenario, which leads to the generation of extracellular deleterious Aβ plaques. Not all steps of APP biogenesis are clear so far, and these questions should be addressed in future studies. AD is a complex chronic disease with many factors that contribute to disease progression.

## 1. Introduction

Alzheimer’s disease (AD) is a severe neurological disorder and the most common type of dementia across the world. According to Alzheimer’s Association, AD contributes to 60–80% of all dementia cases worldwide. As estimated, in 2023, there will be 6.7 million people who are 65 years old or older living in the United States with Alzheimer’s disease [1]. It is predicted that a dramatic elevation of AD pathology will occur in the future, with 13.85 million Americans affected by Alzheimer’s dementia and 152 million affected around the world by the year 2050 [2,3]. In 2019, the World Health Organization (WHO) reported USD 1.3 trillion as the dementia cost around the world, including care expenses from family members and friends who do not fall into the category of professional caregivers and medical personnel. A huge number of current AD patients, their dramatic increase with an aging population in the near future, and the devastating economic costs put pressure on governments to address these issues through new policies for medical care providers to find efficient ways to treat patients and for the scientific community and pharmacologists to discover the mechanism of this disorder, developing markers for its early detection and finding new potential effective treatment and the disease cure. However, despite the fact that intensive studies and significant funding for Alzheimer’s disease research have been undertaken, no breakthrough discovery has been made regarding the mechanism, and many promising therapies have failed; currently, only a few pharmacological treatments have received approval or are under consideration by the FDA, providing only mild improvement in patients [4]. Thus, the significance of the study related to AD is obvious.

Alzheimer’s disease is represented in two forms—familial (inherited) and sporadic. Familial AD is the autosomal-dominant form of the disease and is characterized by relatively early onset under the age of 65, contributing to around 1% of all cases [1]. The sporadic form usually develops after 65 years and, therefore, is referred to as late-onset Alzheimer’s disease (LOAD). It is the most common form of AD. Familial and sporadic cases are triggered by mutations in different genes (discussed in detail below) or by alternative mRNA splicing [5]. There are several studied cases with a very early onset of AD reported, suggesting the increasing number of affected people, even in the younger generation [6,7,8,9]. With the age of disease decreasing and the general population getting older, the development of functional treatment, preventive medicine, and effective diagnostics stay in the focus of attention and is the most wanted.

The clinical picture of Alzheimer’s disease is identical for inherited and familial cases. It comprises memory loss, decreasing thinking skills and solving problems, and the inability to cope with daily tasks. This functional decline is accompanied by changes in personality and behavior and withdrawal from social life and work. Finally, in the late stages, patients are fully dependent on caregivers or special facilities. Alzheimer’s disease affects not only patients diagnosed with this disorder but also their families, with the patients being a large burden in many ways. The progression of this disease usually takes years and starts much earlier than the first symptoms can be detected. Biological changes, such as the presence of specific biomarkers in patient samples or the accumulation of pathological hallmarks, can help to diagnose the disease at the so-called preclinical or pre-symptomatic stage [1,10]. Mild cognitive impairment represents the next stage of the disease progression, characterized by mild symptoms without much interference with daily tasks. The final stage is Alzheimer’s dementia, which can also be in mild, moderate, or severe, causing minor to drastic interference with everyday life. 

Alzheimer’s disease has a strong association with genetics and cellular mechanisms, yet it is a chronic and complex disorder where additional risk factors contribute to disease onset and progression. Genetics and age are the strongest and nonmodifiable risk factors. Health factors (heart and blood vessel conditions, hypertension, and diabetes) and behavior factors (diet, physical activity, level of education, and cognitive engagement) are mixed together in an intricate interplay where the variables depend on each other. 

## 2. The Genetics of Alzheimer’s Disease

The molecular basis of Alzheimer’s disease has been studied for decades. Among the hallmarks of neurodegenerative disorders, the most recent data define not only the aberrant aggregation of proteins but also the dysfunction of neuronal networks, defective energy metabolism, abnormalities in the cytoskeleton, and alterations to protein and metal homeostasis, as well as declining memory, language, and thinking abilities [11]. 

The most known and studied molecular marker of AD is the accumulation of extracellular plaques built up by amyloid β protein (Aβ) and intracellular neurofibrillary tangles (NFTs) formed by hyperphosphorylated tau-protein in brain neurons. To date, several hypotheses of Alzheimer’s disease onset are being discussed in the field. The major hypothesis implicates the defective cleavage of amyloid precursor protein (APP) and the consequent amyloid beta plaque formation as a predominant basis for Alzheimer’s disease, giving rise to a downstream cascade that leads to tau-pathology [12]. However, nowadays, there is a tendency to show the interplay between these two factors [13,14]. 

Although aggregated Aβ and tau are the major characteristics of AD on the microscopic level, the molecular pathology of the disease is not limited to only these two proteins. There is a plethora of genes associated with a higher risk for Alzheimer’s disease. Genome-wide association studies (GWASs) help to identify novel mutations in those genes related to the sporadic form of the disease. This topic has been in the research field for years, widening the list of potentially pathogenic mutations and confirming the genetic complexity of Alzheimer’s disease [15,16,17]. There has been progress in genetic screens that are linked to other genes, including *APOE*, *TREM2*, *SORL1*, and *ABCA7*, with the disease [18,19]. Recent studies identified 75 loci for AD (42 of them were new, and 33 were previously found) [20]. Some of these newly identified genes may regulate APP recycling in the endosomal system and modulate APP metabolism by influencing lipid metabolism and inflammation [21,22,23]. 

*APOE4*, a variant of the *APOE* gene, is associated with a high risk of the development of the sporadic form of AD, but the reason for such effect is still not clear. Apolipoprotein E, which is encoded by this gene, regulates lipoprotein uptake and interferes with lipid transport and lipid metabolism in the brain. Defects in *APOE4* lead to the common pathological characteristics of AD, such as mitochondrial dysfunction, changes in synaptic plasticity, and neuroinflammation [24]. Transcriptomic analyses of APOE4 neurons, astrocytes, and microglia-like cells (derived from induced pluripotent stem cells—iPSC) revealed many differentially expressed genes. Notably, in APOE4 neurons, the production of Aβ is increased, as well as the number of endosomes, where major Aβ generation takes place [25]. With elevated neuronal Aβ production, Aβ uptake by astrocytes is compromised, leading to an increase in extracellular amyloid deposition. These events are accompanied by the activation of an inflammatory response in microglia-like cells and the upregulation of proinflammatory genes [26,27]. The removal of the APOE4 allele in a mouse model leads to a decrease in another AD hallmark, hyperphosphorylated tau and tau-associated neurodegeneration in microglia. It indicates that APOE4 affects tau pathology [26,28,29]. Changes in cholesterol metabolism were also observed. Another study using transcriptomic analysis demonstrated alterations in lipid metabolism in APOE4 astrocytes and microglia, resulting in increased cholesterol synthesis in combination with high cholesterol accumulation in lysosomes, suggesting defects in cholesterol turnover in these cell types; however, this was only in humans [30]. Eventually, an oversupply of cholesterol by astrocytes promotes amyloidogenic APP processing in neurons due to the increased formation of lipid rafts, which APP is associated with [31]. 

Familial forms of AD are connected to the defective proteins involved in the generation of Aβ and are caused by mutations in *PSEN1*, *PSEN2*, or *APP* genes. For the *APP* gene, 25 mutations were described as pathogenic [32]. For *PSEN1* and *PSEN2*, there are around 200 different pathogenic mutations that have been identified as contributing to disease development [33,34]. The *PSEN1* and *PSEN2* genes encode the proteins presenilin 1 and presenilin 2, respectively. Both these proteins modulate the activity of γ-secretase, a membrane-associated complex responsible for the cleavage of different proteins, including APP. It was established that mutations in *PSENs* affect γ-secretase activity through the destabilization of the enzyme-substrate complex. In APP processing, the production of longer Aβ peptides is what stimulates amyloid generation and shifts the balance towards Aβ accumulation [35]. Additionally, it was suggested that *PSENs* mutations trigger pathological alterations in mitochondrial metabolism, which is one of the cellular hallmarks of AD [36]. Mutations in *APP* contribute to AD by increasing the production of the most toxic Aβ42 peptides or through stimulating Aβ aggregation but not through the alterations of APP function [34]. 

## 3. Early Biogenesis of Amyloid Precursor Protein

Despite extensive research into APP biology, especially its processing, the early steps of APP biogenesis are still unknown. Generally, newly synthesized proteins are marked with specific targeting signals for the final protein destination. Depending on these signals, the proteins are transported to different organelles such as the endoplasmic reticulum (ER), Golgi apparatus, plasma membrane, nucleus, mitochondrion, endosomes, lysosomes, or peroxisomes. APP is located in the plasma membrane and other membrane organelles. Thus, it must undergo certain trafficking to reach these subcellular locations. Here, we discuss the general protein trafficking pathway in eukaryotic organisms and analyze its relevance to APP biogenesis.

In general, secretory and membrane proteins follow a specific path during their biogenesis. For proper folding and transport, they are targeted to the endoplasmic reticulum with the assistance of the signal recognition particle (SRP), which is a major route of protein transport in eukaryotes [37,38]. SRP is a ribonucleoprotein complex that is able to bind signal peptides and ribosomes, and is able to transport its cargo to the SRP receptor (SR) in the ER membrane. Mutations in the SRP subunits are associated with multiple human diseases [39]. SRP recognizes a specific part of a polypeptide emerging from the ribosome exit tunnel during translation. This cleavable N-terminal region of secretory proteins is known as a signal peptide; its properties and features were originally described in Dr. G von Heijne’s works [40,41,42]. It was shown that signal peptides do not have amino acid sequence homology; instead, they have common physico-chemical properties, including a stretch of hydrophobic amino acids in the central part. The importance of this signal peptide’s parameters was highlighted in several studies [43,44,45,46]. The defective signal peptide of preprolactin (PPL) does not allow for normal interaction between SRP and the nascent chain of PPL, triggering a specific mechanism of mRNA degradation, named regulation of aberrant protein production (RAPP) [44]. RAPP is one of the protein quality control mechanisms in eukaryotes, and it is activated when SRP cannot bind the nascent chain and the targeting of secretory and membrane proteins is compromised [47,48]. So far, RAPP has been associated with the degradation of the mRNAs of several different secretory proteins in addition to preprolactin. Thus, it was shown that disease-associated mutations in multiple secretory proteins, including granulin (the protein associated with neurodegenerative disease frontotemporal lobar degeneration or FTLD), activate the RAPP pathway [49,50]. It was also suggested that SRP is involved in alpha-synuclein biogenesis, and RAPP may play a role in Parkinson’s disease [51]. Finally, a deep RNA-seq analysis revealed the connection between the loss of SRP interaction with a signal peptide and various metabolic, immune, and age-related disorders, as well as cancer [52]. It was established that RAPP is a general pathway that controls the quality of SRP-dependent secretory and membrane proteins in the ribosome [52]. However, despite the in-depth studies on the interaction between SRP and ribosome-associated polypeptides and the control of their quality during translation, the fundamental questions of which proteins are SRP-dependent and which proteins are SRP-independent have not been answered yet. 

Similar to many secretory and membrane proteins, APP has an N-terminal signal peptide, which is remarkably hydrophobic. The APP signal peptide consists of 17 amino acid residues, and five of them are leucines, which makes it a potential candidate for being an SRP substrate. The APP signal sequence marks this protein for ER targeting, but it was linked to SRP only indirectly [53] and was briefly discussed as a client for cotranslational targeting [54]. There are few studies in which the early stages of APP biogenesis are the focus of the interest. APP was identified as a client of the SEC61 translocon, one of the main entry gates to the ER [55]. The SEC61 translocon is a protein complex in the ER membrane, to which SRP cotranslationally brings its cargo [56,57]. SEC61 is one of the major entry points to the ER, and it can be engaged with other trafficking partners [58,59]. Thus, it is still to be determined how APP is targeted to the ER and what partners are involved in its transport; this can shed light on early APP biogenesis and its possible effect on Alzheimer’s disease onset. 

## 4. Amyloid Precursor Protein Processing

Amyloid precursor protein is a type I membrane protein. It is encoded by the *APP* gene located on chromosome 21 in humans [60,61,62]. APP is widely expressed in the body, with higher expression in the neuronal tissues in the brain. Differential processing and alternative splicing generate different isoforms of APP in a tissue-dependent manner [63]. The three major variants are APP695, APP751, and APP770, and all of them are capable of producing amyloid beta [64]. Isoforms APP751 and APP770 are mostly present in non-neuronal tissues, while APP695 is predominantly found in neurons and is considered the most toxic. APP functions are diverse and are associated with the neurogenesis and differentiation of neuronal cells, synaptic mechanisms, cell cycle and adhesion, and calcium metabolism [65,66,67]. APP-deficient mice exhibit a shortened lifespan, cognitive and learning impairment, and altered metal homeostasis in the brain regions typically affected by the disease [68,69,70]. 

**Figure 1:**
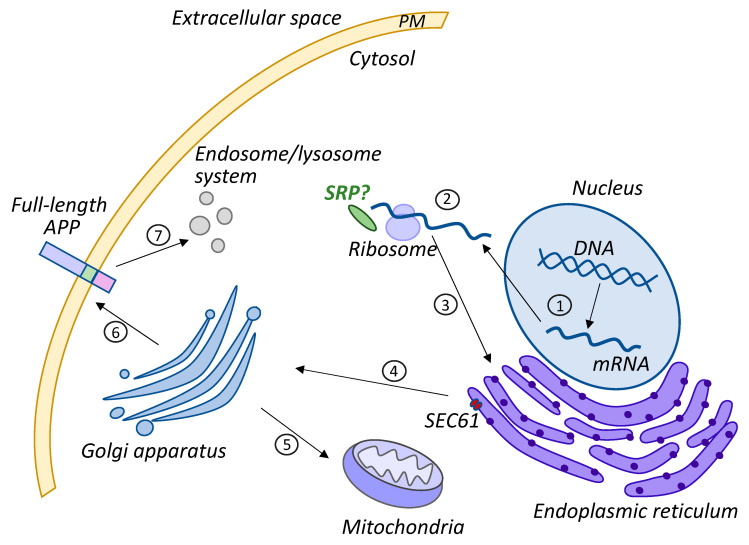
Intracellular APP trafficking. (1) *APP* transcription in the nucleus and mRNA export to the cytoplasm. (2) APP mRNA translation on a ribosome with the assistance of SRP or unestablished targeting factors (no experimental evidence of SRP involvement yet, thus, it is indicated by a question mark). (3) The transport of nascent APP to ER for further biogenesis. (4) The transition to Golgi for post-translational modifications. (5) The transport of full-length APP to mitochondria and the insertion into the mitochondria membrane. (6) The transport of full-length APP to the plasma membrane (PM). (7) The internalization of full-length APP into the endosomal system for further cleavage.

APP processing is a multistep mechanism that involves several cleavage events to release different products. APP biogenesis can be divided into distinct general steps, as shown in Figure 1.

**Figure 2:**
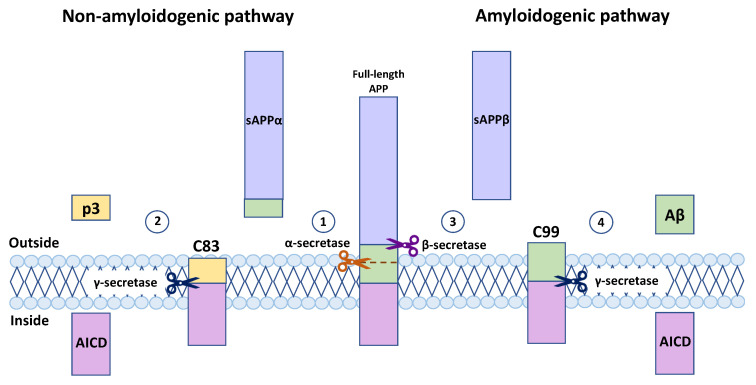
Amyloid precursor protein processing pathways. Full-length APP is inserted into the plasma membrane or intracellular membrane organelles, where it can proceed to the non-amyloidogenic or amyloidogenic pathway. (1) The non-amyloidogenic pathway starts with cleavage by α-secretase, which cuts full-length APP at the Aβ mid-region. This cleavage produces sAPPα and the membrane-bound C83 fragment. (2) The C83 fragment is cleaved further by γ-secretase to release the p3 molecule extracellularly and AICD (amyloid precursor protein intracellular domain) intracellularly. (3) The amyloidogenic pathway starts with β-secretase cleavage, which occurs on the membrane. It produces extracellular sAPPβ and the C99 fragment associated with the membrane. (4) γ-secretase cuts the C99 fragment, and Aβ is released outside of the cell, whereas AICD stays inside.

The vast majority of the studies focus on the late stages of APP processing when the full-length APP is inserted into the plasma membrane or other intracellular membrane organelles and undergoes cleavage events. The cleavage of membrane-inserted APP can follow two pathways—amyloidogenic or non-amyloidogenic (the most common one) (Figure 2). Three secretases play a central role in the late processing of APP: α-, β-, and γ-secretase. The non-amyloidogenic pathway starts with α-secretase releasing the N-terminal extracellular soluble APP domain (sAPPα) and the membrane-attached C83 fragment. α-secretase cuts the middle of the Aβ region; thus, this cleavage prevents the further formation of Aβ. Extracellular sAPPα can mitigate amyloid beta production via the inhibition of β-secretase (BACE1), which is the enzyme responsible for one of the steps in the amyloidogenic pathway of APP processing. Thus, sAPPα stimulates the non-amyloidogenic pathway [71,72]. The reintroduction of sAPPα into APP-depleted mouse models leads to the restoration of a normal phenotype, indicating the pivotal role of the sAPPα fragment in development [73]. 

The first cleavage in the amyloidogenic pathway is performed by β-secretase, also known as β-site APP-cleaving enzyme-1 (BACE1). This cleavage produces the extracellular soluble APP β (sAPPβ) fragment and membrane-bound C99 domain. The importance of BACE1 for the production of aberrant amyloid beta was demonstrated in several studies. Remarkably, the experiments with mouse models for Alzheimer’s disease revealed the complete absence of amyloid beta when BACE1 was silenced [74,75,76,77]. β-secretase is a membrane-associated enzyme with complex trafficking and diverse cellular routs. It is synthesized in the ER in a proenzyme form, which acquires its full activity after several post-translational modifications in the Golgi, including palmitoylation, glycosylation, acetylation, and phosphorylation, which have been shown to be essential for this enzyme to trigger amyloidogenic events [78,79]. After β-secretase insertion into the plasma membrane lipid rafts, it can be extracellularly released; therefore, APP processing by this enzyme rarely occurs on the plasma membrane. Then, this extracellular BACE1 is endocytosed to appear in the endosomes for functioning or to proceed to the lysosomes for degradation. Inside of the cell, BACE1 is mostly located on the membrane of the trans-Golgi network (TGN) and endosomes, where APP processing takes place [80]. Interestingly, initially, the APP from the plasma membrane is internalized through a clathrin-mediated mechanism, whereas BACE1 uses another clathrin-independent mechanism [81]. The optimal pH for this enzyme is 5.5; therefore, the predominant location of the possible APP processing and generation of Aβ is endosomes and lysosomes. Eventually, APP and β-secretase colocalize in Rab5-positive endosomes, where APP is cleaved by a fully active enzyme [82,83]. 

The first cleavage in both pathways results in the formation of the C83 and C99 membrane-bound domains in non-amyloidogenic and amyloidogenic scenarios, respectively. The physiological role of the C83 and C99 fragments is still uncharacterized. Both fragments are substrates for the γ-secretase enzyme complex. The cleavage of C83 or C99 by γ-secretase results in the production of amyloid precursor protein intracellular domain (AICD) in both scenarios, amyloidogenic and non-amyloidogenic. AICD is known as a transcription factor containing the motif YENPTY, facilitating binding to other proteins [84,85,86,87]. AICD has been shown to be one of the regulators of APP processing, promoting intracellular APP trafficking. The APP intracellular domain can be phosphorylated at S655, stimulating the non-amyloidogenic pathway due to directing APP from endosomes with active BACE1 to TGN [88,89,90]. Phosphorylation at the position T668 may promote the amyloidogenic pathway [91], and likely, it interferes with APP intracellular processing [90,92]. Membrane-associated γ-secretase is a complex consisting of at least four transmembrane enzymes—presenilin (PS1 or PS2), presenilin-enhancer 2 (PEN-2), nicastrin (NCT), and anteriorpharynx-defective-1 (APH-1) [93,94]. As mentioned earlier, mutations in *PSEN1* or *PSEN2* genes contribute to the development of familial AD. γ-secretase is not exclusively associated with APP processing. It is implicated in the Notch-pathway and tumorigenesis. There are more than 50 proteins, including E-Cadherin, CD44, and IGF1R (insulin-like growth factor receptor) among γ-secretase’s substrates [95,96]. The location of γ-secretase is not limited to the plasma membrane; it is also located in mitochondria and lysosome membranes, as well as in early and late endosomes [97,98]. The ubiquitous localization of this enzyme complex supports the idea of the highly intricate processing of APP with many subcellular loci available for the potential generation of Aβ. Noticeably, the non-amyloidogenic pathway is predominantly associated with the plasma membrane [99,100], whereas amyloidogenic is connected to the endosomal system [82,83,101]. When C83 is cleaved by γ-secretase, another product, p3, is released into extracellular space in the non-amyloidogenic pathway. To date, the biological role of this molecule has not been established.

Amyloid beta is one of the final products in the amyloidogenic pathway of APP processing. It is a small peptide consisting of 37–43 amino acids, where the Aβ42 isoform is known to be the most deleterious. Aβ peptides can form extracellular soluble oligomers and plaques and insoluble fibrils, which are the main hallmark of Alzheimer’s disease. This accumulation gives rise to pathological events, such as neuroinflammation, cytotoxic effects, and neuronal death. The aggregation of Aβ and its dynamics in laboratory conditions in vitro has been carefully investigated through various methods, including cryo-electron microscopy, atomic force microscopy, nuclear magnetic resonance, electron paramagnetic resonance, and X-ray [102,103,104,105,106]. Aβ peptides can build up in a different fashion to form diverse structures of β-sheets, depending on the arrangement of monomers and the orientation of β-strands and β-sheets [107]. An intriguing phenomenon of aggregated Aβ peptides was observed in several studies. Amyloids consisting of 2–12 monomers are considered to possess the highest level of toxicity, whereas longer forms can interact with their shorter counterparts to “isolate” them, reducing the harmful effects. Therefore, the aggregation of Aβ, despite being a main pathological signature of the disease, can actually help cells to survive via the mitigation of cytotoxic effects [108,109]. Another deleterious effect of Aβ accumulation is the disruption of the plasma membrane followed by changes in calcium (Ca^2+^) flux. The pore-forming hypothesis is still controversial; however, growing evidence indicates that, indeed, Aβ oligomers are inserted into the plasma membrane where they form Ca^2+^-permeable pores and disrupt calcium homeostasis, which also leads to neuronal damage and cell death [110,111].

## 5. Amyloid Precursor Protein and Mitochondria

It has been known and investigated for years that mitochondria are connected to amyloid precursor protein and Alzheimer’s disease. This field can be divided into three major research questions: (1) how is APP transported to mitochondria, and where is it localized? (2) What is the role of APP in mitochondria, and how is mitochondrial metabolism affected during the disease? (3) Can mitochondria be a therapeutic target for AD treatment?

As of today, mitochondrial dysfunction is one of the pathological hallmarks of Alzheimer’s disease; nevertheless, this condition is present in the majority of neurodegenerative disorders [112,113]. In addition to the plasma membrane, APP is transported to mitochondria due to a signal sequence resembling a mitochondrial-targeted signal [114,115]. Initially, although APP was found in the mitochondria of AD patient brain samples, a recent study showed APP presence in both healthy and pathological brains [116,117]. Mitochondrial dysfunction was attributed to Alzheimer’s disease, causing defects in metabolism, protein maturation in mitochondria, energy production, oxygen consumption, and mitochondrial calcium homeostasis [118,119,120]. Multiple attempts were made to assess the changes in mitochondrial metabolism with the overexpression of wild-type APP or its mutated forms. The results vary from one cell line to another as well as between study groups; therefore, there is no consensus about APP effects in mitochondria. A comprehensive review describes, in detail, the results of recent studies on how APP effects mitochondria in in vitro and in vivo models [121]. Mitochondria metabolism seems an appealing target for potential AD therapy. Cell replacement therapy with MSCs or MSC-conditioned media has the potential for a reduction in oxidative stress and the restoration of normal mitochondrial function in a mouse model [122]. Exploiting nanoparticles for targeting mitochondria was also investigated. MSCs-derived extracellular vesicles (EVs) with tyrosine phosphatase-2 (SHP2) deliver SHP2 to the brain, where it induces mitophagy and helps with the clearance of aberrant proteins [123,124]. 

## 6. Alzheimer’s Disease Is a Complex Disorder

**Figure 3:**
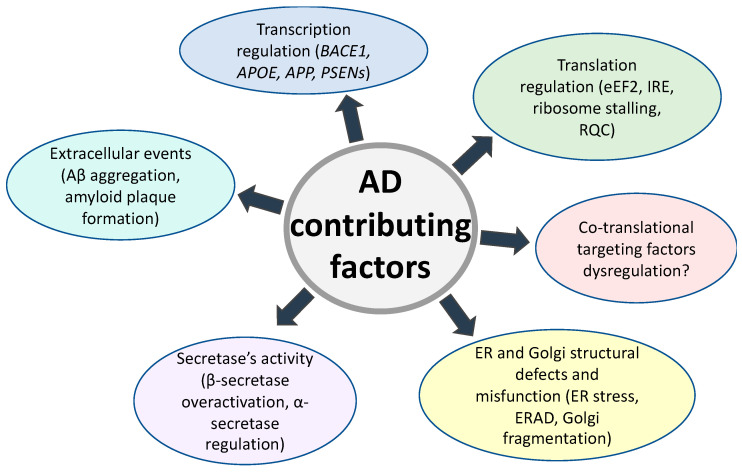
Alzheimer’s disease contributing factors.

Alzheimer’s disease pathology is triggered by genetic factors, such as inherited mutations in familial cases or sporadic mutations with a connection to age. However, there are other factors affecting the disease progression and severity of the symptoms. AD contributing factors are spread over all parts of APP biogenesis (Figure 3). 

Versatile evidence indicates that Alzheimer’s disease emerges from an imbalance between amyloid plaque accumulation and its degradation. The regulation of amyloid β production starts on the level of transcription. Autophagy is the main cellular mechanism for the clearance of aggregates and aberrant proteins. PPARA/PPARα (peroxisome proliferator-activated receptor alpha) regulates the gene expression of autophagy, with lipid and glucose metabolism genes serving as some of the central regulators for mitochondrial function [125]. The pharmacological targeting of transcription factor PPARA activates autophagy in human microglial and glioma cells expressing APP, which leads to the partial removal of amyloid plaques and causes a shift towards Aβ clearance through transcriptional regulation [126]. A shift towards amyloid β production can be triggered by the regulation of genes directly involved in APP processing. For instance, *BACE1*, the gene that encodes β-secretase, has several transcription factor-binding sites that allow for the regulation of this gene by multiple transcription factors, for example, peroxisome proliferator-activated receptor gamma (PPARγ), NF-κB, specificity protein 1 (SP1). PPARγ is a nuclear transcription factor that reduces the activity of the *BACE1* promotor when overexpressed. In AD patient, samples of a lower level of PPARγ was detected, suggesting the overactivation of *BACE1* [127]. NF-κB regulates *BACE1* expression differently under different conditions, such as lowering BACE1 expression in physiological conditions but promoting Aβ generation in pathology [128]. SP1 is one of the first identified regulators for *BACE1*, working as an activator for β-secretase expression and also interacting with NF-κB [129,130]. The transcription of *APOE* can be upregulated by cyclic AMP (cAMP), retinoic acid (RA), PPARγ, and Aβ itself. In the case of *APOE* transcriptional regulation via Aβ, it can be considered as a neuroprotective mechanism since ApoE helps prevent against cytotoxicity [131]. 

*APP* transcription can be activated in different cell types by heat-shock factor 1 (HSF-1), NF-κB, and Rac1 [128]. *PSEN1* transcriptional regulation has been studied more than *PSEN2* and can be controlled by diverse transcription elements (Ets, ZNF237, cAMP-responsive element-binding protein, and p300) and chromatin modifications [132,133,134]. 

The activity of α-secretase is also subject to regulation, leading to changes in the balance of amyloid production. The protease furin effectively promotes the non-amyloidogenic pathway and the production of sAPPα, which, in turn, further stimulates this pathway. When furin is inhibited, the level of sAPPα is decreased (when the APP level is not changed), suggesting amyloidogenic pathway activation [135]. Remarkably, a recent study revealed an interplay between iron overload in neurodegeneration and the downregulation of furin, leading to elevated production of amyloids [136]. 

Defects in other stages of APP processing may also play a significant role in Aβ accumulation and aggregation. It was shown that alterations to APP mRNA translation can happen in the initiation and elongation steps. Elongation factor eEF2, when phosphorylated, slows down protein synthesis and leads to ribosome stalling. In AD mouse models, the phosphorylation of eEF2 is enhanced, suggesting the involvement of this elongation factor in AD pathology development [137]. Another mechanism that was shown to alter APP mRNA translation is iron-dependent. It was demonstrated that patients with neurodegenerative disorders, such as Alzheimer’s or Parkinson’s disease, have an elevated level of iron in the brain [138]. Iron toxicity is connected to the generation of reactive oxygen species in the brain following oxidative stress and neuronal death [139]. The iron-responsive element (IRE) of APP mRNA was identified and shown to regulate APP protein expression. When iron is chelated in a neuroblastoma cell line, APP protein synthesis is drastically decreased, demonstrating the involvement of iron in the regulation of APP translation [140]. If ribosomes with an APP nascent chain are stuck in the ER during translation, this activates ribosome-associated quality control (RQC), triggering a down-stream cascade of reactions. Abnormal RQC causes endolysosomal misfunction, which leads to the formation of an amyloid plaque core intracellularly [55]. Yet, as mentioned before, the APP targeting factors are still unestablished, and this can be another aspect of APP processing that might contribute to AD development. 

The prevalent part of APP maturation takes place in the ER and Golgi, where post-translational modifications occur; therefore, these subcellular locations are important for physiologically normal APP biogenesis. A plethora of studies have focused on ER stress and its connection to Alzheimer’s disease. The unfolded protein response (UPR) is a protein quality control mechanism associated with ER. During mild ER stress, this mechanism is highly functional and beneficial for cells since it works for balancing ER and protein homeostasis [141]. But under severe stress conditions in AD, the UPR turns maladaptive and stimulates apoptosis, increasing neuronal death [142]. Another connection between APP biogenesis and ER was made through ER degradation-enhancing α-mannosidase-like protein 1 (EDEM1), a targeting factor for misfolded ER proteins. It targets aberrant proteins for degradation in the ER-associated protein degradation (ERAD) pathway [143]. In cell cultures expressing APP, EDEM1 promotes APP targeting from the ER to the cytoplasm, where ERAD takes place. It stimulates the proteosome degradation of APP inside the cell and leads to a consequent decrease in Aβ40-42 production [144]. Golgi fragmentation was reported in AD pathology cases; however, the primary reason and consequence in the APP-Golgi relationship is still poorly understood. It was suggested that despite this, Golgi defects appear as a consequences of AD pathology in the early stages, and these defects also enhance amyloid formation and stimulate the amyloidogenic pathway [145]. The fragmentation of the Golgi apparatus is due to the phosphorylation of Golgi structural proteins (GRASP65), which happens through the Aβ-associated activation of cyclin-dependent kinase-5 (cdk5) [146]. 

## 7. Alzheimer’s Disease Diagnostic and Treatment

Nowadays, technical progress has allowed for the diagnosis of Alzheimer’s disease, even in the preclinical stage. An inadequate level of Aβ and hyperphosphorylated tau protein can be detected in cerebrospinal fluid (CSF). Positron emission tomography (PET) allows for the detection of accumulated amyloids and tau tangles in the brain. Recently, a new diagnostic technique was introduced. Blood-based biomarkers (BBMs) for Alzheimer’s disease screening have several advantages, such as the simplicity of the test and its performance (blood tests can be carried out in any medical facility, whereas CSF analysis or PET can only be conducted in specialized clinics), lower cost, and additional biomarkers for neurodegeneration (neurofilament light chain (NfL) and glial fibrillary acidic protein (GFAP)) can be detected [147,148,149]. Several other methods, such as computed tomography (CT) or magnetic resonance imaging (MRI), may be used to refute Alzheimer’s disease pathology or to support other possible diagnoses. 

There are many potential options for the treatment of Alzheimer’s disease, which are broadly being explored to this day. One of the most promising fields is antibody-based therapy to target Aβ. Several developed immunotherapy compounds have already entered clinical trials; however, many of them failed. By the end of 2022, there were four antibody-based therapeutic agents undergoing the final clinical phase: aducanumab, lecanemab, gantenerumab, and donanemab. These are monoclonal IgG1 antibodies with an affinity to aggregate amyloid beta forms [150]. In spite of high hopes for finding a curative medicine, it is too early to say if some of the suggested options may revolutionize Alzheimer’s disease treatment. 

Another treatment option that has become widely explored is stem cell therapy. Mesenchymal stem cells (MSCs), neural stem cells (NSCs), and embryonic stem cells (ESCs) are used for transplantation into AD mouse models to evaluate their potential curative effect on neurodegenerative pathology. Among the common effects between these different MSC lines, there has been an increase in synaptic plasticity, mitigation of inflammatory response, improved short-term memory and learning abilities, and cognitive improvement [151,152,153,154,155,156]. 

Since the sporadic form of Alzheimer’s disease has a strong association with aging, anti-aging therapy is considered another approach for AD treatment, which has been actively investigated. Several existing anti-aging drugs are under investigation with nanoparticle-based delivery agents in animal models [157,158]. Nanoparticle-based treatment delivery is believed to be effective because nanoparticles can penetrate the blood–brain barrier (BBB) efficiently and perform targeted delivery with a lower chance of crossing peripheral circulation.

## 8. Conclusions

Alzheimer’s disease is a complex chronic disorder where genetic defects are enhanced by age, other health conditions, and environmental factors. The investigation of the genetic features of AD using modern technological approaches has allowed for a broader picture of the diagnostics of the disease. Detailed studies on the molecular biology of APP and all the related products, including secretases, have helped determine the relationship between them and how they affect the amyloidogenic process. Despite decades of research on Alzheimer’s disease and APP, we are still far from a complete understanding of its biological basis. Many questions about APP biogenesis, especially the early steps, interacting partners, APP’s role in mitochondria, and the potential therapeutic targets, must be addressed in future studies. 

## Data Availability

Not applicable.
